# Pregnancy outcomes of normal karyotype fetuses with increased nuchal translucency

**DOI:** 10.22088/cjim.14.4.732

**Published:** 2023

**Authors:** Shirin Niroomanesh, Narges Nadimzadeh, Fatemeh Rahimi-Sharbaf, Mahboobeh Shirazi, Fatemeh Golshahi, Behrokh Sahebdel, Elham Feizabad

**Affiliations:** 1 Department of Obstetrics and Gynecology, Yas hospital, Tehran University of Medical Sciences, Tehran, Iran

**Keywords:** Nuchal translucency, Fetal abnormalities, Preterm labor, Preeclampsia, IUGR, Hydrops fetalis.

## Abstract

**Background:**

Although the association between first-trimester increased nuchal translucency (NT) and chromosomal defects is well-defined, there is little knowledge about the importance of increased NT in fetuses with normal karyotypes. Hence, this study was designed to evaluate outcomes of normal karyotype fetuses with increased NT.

**Methods:**

This observational retrospective study was conducted on 720 pregnancies with increased NT (>3mm) at 11 to 13 weeks of gestational age in Yas Hospital, Tehran, Iran, from 2016 to 2020. Study outcomes were incidence of preeclampsia, intrauterine growth restriction) IUGR (, preterm labor, fetal anomaly, cardiac malformations, hydrops fetalis, abortion, and intrauterine fetal death (IUFD) in pregnancies without evident chromosomal karyotype abnormalities.

**Results:**

Out of 720 participants who underwent karyotyping in the study center, 523 fetuses had normal karyotype results. In the pregnancies assessed for outcome, 357 (68.3%) reached live birth, 104 (19.9%) aborted, and 62 (11.9%) resulted in IUFD.

Study outcomes including preeclampsia (<0.001), IUGR (<0.001), preterm labor (<0.001), fetal anomaly (<0.001), cardiac malformations (<0.001), hydrops fetalis (0.017), abortion (<0.001), and IUFD (<0.001) resulted in a statistically significant difference in the distribution of cases across NT intervals.

**Conclusion:**

This study demonstrated adverse pregnancy outcomes increased with increasing NT despite normal karyotyping. Hence, tight observation of mothers and fetuses should be done in normal karyotype fetuses with increased NT.

Nuchal translucency (NT), as a non-invasive, affordable, and widely accessible ultrasound marker in prenatal screening, is a fluid accumulation behind the fetal neck in the first trimester of pregnancy (1). The normal thickness of NT is dependent on gestational age (GA) and is considered alongside the crown-rump length and the 95th reference value has been reported to be 1.8-2.35mm during 11 to 13th gestational weeks in the Persian population (2). Since in 80% of aneuploidy embryos, the thickness of the NT is increased, when a pregnancy with an abnormally higher than normal NT thickness is encountered, chorionic villus sampling (CVS) or amniocentesis is recommended to determine the fetal karyotype (3, 4). 

The most common chromosomal abnormalities seen with high fetal NT are trisomy 21, 18, and 13. In addition, cardiovascular abnormalities show an increase in NT in both normal and abnormal fetuses in terms of chromosomes, so heart defects are one of the most common causes of high fetal NT (5). 

On the other hand, in fetuses with increased NT and normal karyotypes, a wide range of fetal malformations, genetic syndromes, structural abnormalities, and adverse perinatal outcomes were reported (6). This complication is directly related to the amount of NT in the first-trimester examination and it seems the higher NT level has a greater risk (7). Although the association between first-trimester increased nuchal translucency (NT) and chromosomal defects is well-defined, there is little knowledge about the importance of increased NT in fetuses with normal karyotypes. Several studies (5, 7-9) have questioned the value of increased NT, without karyotyping abnormality. Hence, this study was designed to evaluate outcomes of normal karyotype fetuses with increased NT.

## Methods

This observational retrospective study was conducted on pregnancies with increased NT thickness at 11-13 weeks’ gestational age (GA) referred to the prenatal clinics of Yas Hospital, Tehran, Iran, from 2016 to 2020. The study was approved by Tehran University of Medical Sciences Ethics Committee (IR.TUMS.MEDICINE.REC.1400.1194). Inclusion criterion was pregnant women with NT values greater than 3mm in the established 11 to13 weeks of GA and who underwent CVS or amniocentesis for karyotyping evaluation. The exclusion criterion was incomplete medical records.

 All ultrasound screening test was done by one expert perinatologist through the PHILIPS Infiniti 70 ultrasound machine equipped with a convex trans-abdominal 6-9 MHz probe. Clinical data of the patients were gathered from medical records of the patients during their hospital stay for karyotyping and/or delivery. Study outcomes were incidence of preeclampsia, intrauterine growth restriction (IUGR), preterm labor, fetal anomaly, cardiac malformations, hydrops fetalis, abortion (pregnancy losses occurring before 20 weeks of GA), and intrauterine fetal death (IUFD) (pregnancy losses occurring after 20 weeks of GA), in pregnancies without evident chromosomal karyotype abnormalities.All the statistical analyses were done using Statistical Package for the Social Sciences (SPSS) Version 24.0 (IBM, New York, USA). A p-value< 0.05 was considered statistically significant. The data variables were analyzed at the descriptive level in which descriptive statistics (chi-square test) were used to assess the distribution of adverse outcomes and complications in pregnancies. The standardized residual method was used to further analyze the distribution of variables across NT values and evaluate the difference of incidence of complications across NT value intervals, as described by Beasley and Schumacker (10). 

## Results

 Out of 720 participants who underwent karyotyping in the study center, 523 fetuses had normal karyotype results. The karyotype results of the other participants included Turner syndrome (n= 20), triploid (n=17), trisomy 13 (Patau syndrome, n=27), trisomy 18 (Edwards’ syndrome, n=24), trisomy 21 (Down’s syndrome, n= 97), and other uncategorized patterns (n=12). In the pregnancies assessed for outcome, 357 (68.3%) reached live birth, 104 (19.9%) aborted, and 62 (11.9%) resulted in IUFD (table 1). Of study cases, 182 (39.5%) had preterm labor, and 90 (19.7%) developed preeclampsia. Fetal anomaly was observed in 43 fetuses (6.0%), hydrops fetalis in 75 (10.4%) fetuses, and cardiac malformations in 69 (9.6%) fetuses (table 1). 

**Table 1:** Maternal age and rate of pregnancy complications and adverse outcomes in the study population (n=720)

**Variables**	
**Maternal age (Mean±SD)**	33.4 ± 6.74
**Karyotype results (%)**	
Normal	523 (72.6%)
Turner	20 (2.8%)
Triploidy	17 (2.4%)
Trisomy 13	27 (3.8%)
Trisomy 18	24 (3.3%)
Trisomy 21	97 (13.5%)
Other	12 (1.7%)
**Pregnancy outcome (%)**	
Live birth	357 (68.3%)
Abortion	104(19.9%)
IUFD	62 (11.9%)
Preterm labor (%)	182 (39.5%)
Preeclampsia (%)	90 (19.7%)
Torch infection (%)	16 (2.2%)
Fetal anomaly (%)	43 (6.0%)
Hydrops fetalis (%)	75 (10.4%)
Cardiac malformations (%)	69 (9.6%)

IUGR: Intrauterine growth restriction; IUFD: Intrauterine Fetal Demise

Analysis of distribution of pregnancy complications across NT intervals revealed even mildly increased NT thickness is associated with an adverse pregnancy outcome and all of the evaluated conditions including preterm labor, preeclampsia, IUGR, abortion, IUFD, hydrops fetalis, fetal anomalies, and cardiac malformations resulted in a statistically significant difference in distribution of cases across NT intervals (table 2). 

**Table 2:** Association of pregnancy complications and increased nuchal translucency in pregnancies with normal karyotype results (n=523)

**Disease/Condition**	**Cases per nuchal translucency interval (mm)**							**P-value**
	**3 - 3.5**	**3.5 - 4**	**4 – 4.5**	**4.5 - 5**	**5 – 5.5**	**5.5 - 6**	**>6**	
**Preeclampsia**	14	8	16	8	4	3	1	<0.001
**IUGR**	31	15	19	14	8	4	2	<0.001
**Preterm labor**	44	15	23	16	8	4	3	<0.001
**Fetal anomaly**	2	18	8	4	0	0	1	<0.001
**Cardiac malformations**	1	33	17	3	0	0	1	<0.001
**Hydrops fetalis**	2	6	6	0	2	1	0	0.017
**Abortion**	1	56	39	6	1	1	0	<0.001
**IUFD**	0	6	46	9	0	0	1	<0.001

IUGR: Intrauterine growth restriction; IUFD: Intrauterine Fetal Demise.

## Discussion

This study demonstrated adverse pregnancy outcomes including preeclampsia (<0.001), IUGR (<0.001), preterm labor (<0.001), fetal anomaly (<0.001), cardiac malformations (<0.001), hydrops fetalis (0.017), abortion (<0.001), and IUFD (<0.001) increased with increasing NT despite normal karyotyping. Rajaei et al. examined the increase in NT and normal karyotype. It is seen in 80% of fetuses with chromosomal abnormalities and in 5% of fetuses with normal karyotype. With an increase in NT, even if the karyotype is normal, the incidence of structural defects, genetic syndromes, abortion, and perinatal death increases (11). Rahbar et al. (2012) in a study investigated the relationship between the size of the transparent space behind the fetal neck and the adverse consequences of pregnancy. This study was an analytical cohort study of pregnant women aged 11 to 13 weeks (± 6 days) who were screened for NT and divided into two groups according to NT: 748 in the NT group less than 2 and 360 patients in the NT group were greater than or equal to 2, and pregnancy complications including abortion, preterm delivery, preeclampsia and low birth weight, abortion, and intrauterine fetal death were assessed in each group, which resulted in significant association between preterm labor, preeclampsia, abortion, and low birth weight with NT ≥ 2 (12). On the other hand, in a study by Westin et al. on 16,260 pregnant women with normal fetal karyotype, fetal abnormalities, abortion, perinatal death, termination of pregnancy were not reliably distinguished between normal and adverse outcome in fetuses with normal karyotype using NT screening (13).

Furthermore, in a study by Piazze et al., the accuracy and reliability of NT as a predictor of the outcome of adverse pregnancy outcomes was assessed. They demonstrated that an increase in NT was significantly associated with the occurrence of preterm delivery. But no significant relationship was observed between NT and gestational hypertension and intrauterine growth restriction. There was also no statistically significant relationship between NT increase and abortion threat in their study (14). Maymon et al. (2004) in the NT study on embryos with normal chromosome counts showed a significant relationship between increased NT and the incidence of abortion. In other words, the increase in NT was associated with an increase in pregnancy complications, including abortion (15). On the other hand, Hassanzadeh et al. examined the diagnosis of aneuploidy by amniocentesis in high-risk cases screening test in the first trimester of pregnancy. This descriptive-analytical (cross-sectional) study was performed on 121 pregnant women whose Down Syndrome and other aneuploidy screening results were recorded in the first trimester of pregnancy test (11 weeks to 13 weeks and 6 days) for amniocentesis (15 to 20 weeks), in which 10% of the high-risk identified by first trimester screening test were diagnosed as aneuploidy by amniocentesis (16).

In a small study by Sokolov et al. on 71 cases of increased NT, the majority of cases (55, 78%) were not aneuploid and optimal pregnancy outcome was obtained in 40 patients (56% of total, 72% of euploid pregnancies), suggesting that by using NT screening results only, there is a considerable proportion of cases with good pregnancy outcomes despite abnormal NT results (17). 

Westin et al. (2007) also showed supporting evidence that there is no significant relationship between increased NT and intrauterine death in the study of how much the increase in NT increases the risk of adverse pregnancy outcomes in normal chromosomal embryos (13). Furthermore, in a study by Piazze et al. (2007) that assessed NT acted as a predictor of adverse pregnancy outcomes, no significant relationship between NT and gestational hypertension and intrauterine growth restriction was observed. Also, there was no statistically significant relationship between increased NT and the threat of abortion (14). 

Many structural abnormalities and genetic syndromes are associated with an increase in NT (18, 19). Maternal-to-fetal transmitted infections can also increase NT. As such, if cervical edema persists until 16-20 weeks of gestation, screening for congenital infections is recommended (20). In all cases where the NT is increased in the first trimester of pregnancy, an ultrasound is recommended to be performed by an experienced person at 18-20 weeks to examine the fetus abnormally, and if no abnormalities are found, there is still a small risk (2%) of a poor pregnancy outcome for these fetuses (21). 

While this study demonstrated higher risk of complications and fetal anomalies, evidence should be considered in consultation with parents as well as more careful follow-up and more accurate evaluation of these pregnancies. In fetuses with increased NT at 10-14 weeks of gestation, after confirming the normal karyotype, anomalous ultrasound and echocardiography of the fetal heart should be performed to rule out the possibility of structural and cardiovascular abnormalities of the fetus. It is of note to say that, NT values alone may not indicate fetal abnormalities and when the karyotype is normal and most fetuses will grow normally and will not have problems without the need to terminate the pregnancy.

The strong points of this study were its noticeable sample size and using the same and standard criteria in the diagnosis of pregnancy outcomes. This study had some limitations including lack of a control group, one-center study, as well as retrospective nature research. This study demonstrated adverse pregnancy outcomes increased with increasing NT despite normal karyotyping. Hence, tight observation of mothers and fetuses should be done in normal karyotype fetuses with increased NT.
